# The association between anhedonia and quality of life in treatment-resistant depression: effects of a three-month treatment with esketamine nasal spray

**DOI:** 10.1192/j.eurpsy.2026.11412

**Published:** 2026-07-31

**Authors:** I. Marcelli, M. Pepe, E. Specogna, A. Crupi, S. Barbonetti, L. Chisari, L. Balzoni, E. Stella, M. Lanzetta, M. Di Nicola, G. Sani

**Affiliations:** 1 Department of Neuroscience, Section of Psychiatry, Università Cattolica del Sacro Cuore; 2 Department of Psychiatry, Fondazione Policlinico Universitario “A. Gemelli” IRCCS, Rome, Italy

## Abstract

**Introduction:**

Anhedonia is a core and frequently treatment-refractory symptom dimension of treatment-resistant depression (TRD), strongly associated with functional impairment and reduced quality of life. Esketamine nasal spray (ESK-NS) has demonstrated anti-anhedonic effects in real-world clinical settings; however, the extent to which improvement in anhedonia contributes to functional and quality-of-life recovery remains insufficiently explored.

**Objectives:**

This study examined changes in depressive symptoms, anhedonia, and quality of life in TRD patients treated with ESK-NS for three months. In addition, we investigated whether reductions in anhedonia mediate the association between improvements in depressive symptoms and quality of life.

**Methods:**

Fifty patients with TRD (36.3% female; mean age 54.7±12.4 years) receiving ESK-NS for three months were retrospectively included. Depression severity was assessed using the Montgomery–Åsberg Depression Rating Scale (MADRS) and the Beck Depression Inventory-II (BDI-II). Anhedonia was evaluated using the anhedonia subscales of the MADRS and BDI-II. Quality of life was measured with the EuroQol-5 Dimensions (EQ-5D). Statistical analyses included descriptive measures (mean ± SD, percentages), repeated-measures ANOVA, and mediation analysis, with significance set at p<0.05.

**Results:**

ESK-NS significantly improved depressive symptoms over the treatment period (MADRS, p=0.006; BDI-II, p=0.002). Anhedonia significantly decreased according to MADRS anhedonia scores (p=0.04), while BDI-II anhedonia scores showed marked improvement at the endpoint (p<0.001). Quality of life improved significantly following three months of treatment (p<0.001). Mediation analysis demonstrated that reductions in anhedonia significantly mediated the relationship between depressive symptom improvement and enhanced quality of life (Figure 1).

**Image 1:**
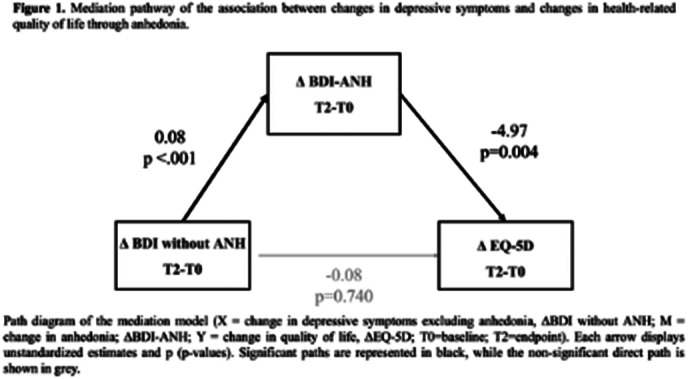

**Conclusions:**

Three-month treatment with ESK-NS significantly improved depressive symptoms, anhedonia, and quality of life in TRD patients. Interestingly, reductions in anhedonia emerged as a key mediator linking clinical improvement to quality of life. These findings underscore the relevance of anhedonia as a central therapeutic target in TRD, highlighting the importance of assessing both symptomatic and quality-of-life outcomes in clinical practice.

**Disclosure of Interest:**

None Declared

